# Using peer advocates to improve access to services among hard-to-reach populations with hepatitis C: a qualitative study of client and provider relationships

**DOI:** 10.1186/s12954-017-0202-x

**Published:** 2017-11-28

**Authors:** Jennifer MacLellan, Julian Surey, Ibrahim Abubakar, Helen R. Stagg, Jenevieve Mannell

**Affiliations:** 1 Oxford University Hospitals Foundation Trust, Oxford, UK; 2 Institute for Global Health, UCL, 30 Guilford Street, London, WC1N 1EH UK

**Keywords:** Narratives, Participation, Self-presentation, Social support, Wellbeing

## Abstract

**Background:**

Peer support programmes use individuals with specific experiences to improve engagement and outcomes among new clients. However, the skills and techniques used to achieve this engagement have not been mapped. This potentially restricts the development and replication of successful peer advocate models of care. This study explored how a group of peer advocates with experience of homelessness, alcohol and drug misuse made and sustained relationships with their client group. For the purposes of this project, the client group were located among a hepatitis C-positive cohort of people who have a history of injecting drug use and homelessness.

**Methods:**

Five self-selecting advocates gave a narrative interview lasting 40–90 min. These interviews were double transcribed using both thematic analysis and narrative analysis in order to triangulate the data and provide a robust set of findings about the unique skills of peer advocates in creating and sustaining relationships with clients from hard-to-reach populations.

**Results:**

Peer advocates build rapport with clients through disclosing personal details about their lives. While this runs counter to assumptions about the need to maintain distance in client-patient relationships, the therapeutic benefits appear to outweigh the potential costs of this engagement.

**Conclusion:**

We conclude the therapeutic benefits of self-disclosure between peer advocates and their clients offer a moral grounding for self-disclosure as a means of building relationships with key hard-to-reach populations.

## Background

Peer support programmes involve individuals with previous patient or challenging social experiences to improve client engagement and clinical outcomes. This can range from informal visits and sharing of experiences to formal appointments focused on information giving and support in relation to the intervention. This programme design has expanded rapidly across disciplines from its origin in mental health, with particular success among marginal and hard-to-reach groups. Examples include working with low-income mothers to increase breastfeeding rates and duration [1–3], middle-aged men with chronic disease in weight loss or diabetes management programmes [4–6], those with stigmatised disease such as human immuno-deficiency virus and tuberculosis to support medication adherence and living well for those who are newly diagnosed [7–10], and individuals with substance abuse to support rehabilitation and negotiation of social and health services [11, 12].

Programme evaluations frequently identify the relationship between the client and the peer as the key component of the intervention’s success [5, 9, 13–15]. This relationship is proposed as originating from a shared understanding of the challenges of the client’s situation from the experience of the peer and fosters trust, understanding and empathy within the relationship. Jacobson [16] names this shared understanding as ‘connectedness’, crediting peer effectiveness to this construct. However, the components of achieving this connection are not disclosed, limiting the ability for standardised guidance in training and thus accreditation of peer support programmes [13].

Within a healthcare context, hard-to-reach groups are defined by their difficulty to engage with the rigid, hierarchical structure of the profession-led health service. The system has been described as inflexible and judgemental, with individuals required to learn the role of ‘patient citizens’ to negotiate their entitlement to treatments and therapies [17]. Although peer advocates (PAs) have no power in the hierarchy of this profession-led institution [18, 19], it is their unique location between the institution and social world of the client that brings success. Their understanding of how the healthcare system operates, partnership with providers and appreciation of perceived access constraints from the client’s experiences come together to facilitate the client’s engagement with services [13].

Studies that qualitatively analyse the impact of the PA role on the PA themselves tend to focus on the extent of personal recovery. A metasynthesis of these studies [13] concluded the ability of the PA to engage with other marginalised individuals reflected characteristics of a therapeutic model of care based on the appropriate sharing of their story. This synthesis also uncovered a process of reframing identity by the peer as a result of the reciprocal relationship with the client, while a sense of responsibility underpinned and often sustained the relationship. Sharing of one’s story is often discouraged without strict boundary setting by many peer programmes to maintain the focus of the care episode on the client and their situation. The role of the peer as advocate not therapist is also emphasised. Therefore, if sharing their story is key to the effectiveness of the peer, how can it be done in a safe, effective way that enables the creation of a supportive, positive relationship for both peer and client. This study aimed to fill this evidence gap by exploring how PAs achieve their connections to facilitate the wellbeing and health service engagement of their client. Since the experiences of the PAs would act as the data for this project, we conducted in-depth interviews with a group of PAs working with marginalised populations.

## Methods

This qualitative study of peer advocates was embedded within the HALT: Hepatitis study, a randomised controlled trial (ISRCTN24707359) of a peer intervention for improving patient engagement with National Health Services across London. Within the study, the PA role was to engage with referred clients, support and advocate for them through the appointment process and generally within peripheral services. The rationale for this design lies in the recognition of the factors that put people at risk of hepatitis C infection (injecting drug use, substance misuse, chaotic lifestyle) and can create barriers to diagnosis and treatment adherence. This is in addition to the stigma and preconceptions surrounding hepatitis C infection and eligibility for treatment. Ethical approval for this qualitative study was granted by the City Road and Hampstead National Research Ethics Service Committee (14/LO/0408_147989). PAs were invited to participate in the study at the fortnightly peer meeting within the organisation at which they were based and thus were self-selecting. Following written consent, a single narrative interview was conducted by the research nurse experienced in qualitative interviewing, on neutral premises with each of the five PAs lasting between 40 and 90 min. These were recorded and transcribed verbatim. Interview content was kept confidential from colleagues and the PA organisation, with all identifiers removed during write up.

It quickly became clear that narratives were used spontaneously by each participant to illustrate their interactions with clients and health providers. This narrative style of the interview allowed respondents to reclaim the interview process and narrate stories they considered important and relevant, providing significant insight into the perspective and approach of the PAs.

Transcripts were analysed, using structural narrative analysis alongside thematic analysis to track the process of relationship building and maintenance between peer, client and health providers. The underlying assumption of the authors in their approach to this project is that narratives are used by people to construct their reality. This social constructionist epistemology supports the role of narrative to bring coherence to our experiences, while constructing our identity in relation to others [20]. The practice of narrative analysis advocated by Reissman [21] takes this in to account by looking at the work achieved by narratives on an individual level through their structural composition, complemented by the consideration of the context of the story through an interpretation of themes in relation to the wider theoretical literature. This use of two analytical tools was felt to add robustness to the analysis, to be relevant to the story formatting of the interview content, while helping to overcome the potential limitations of a small amount of data.

Following the structural narrative analysis, we returned to the text as a whole and conducted a separate thematic analysis to focus on the context revealed in the stories. Thematic analysis is characterised by searching repeatedly across the data to find patterns of meaning [22]. Analysis of these patterns allows wider theorising about the socio-cultural conditions and structural conditions enabling the construction of the data [23]. Within this project, the theoretical literature was accessed following construction of a thematic map from the data. This was then revisited in light of the theoretical suggestions, resulting in further interpretation and refinement to present two meta-themes, informed by two sub-themes. In line with Reissman’s [21] approach, narratives were then selected to illustrate the developing theoretical arguments emerging out of the analysis and the connection between personal stories and larger social concepts.

## Results

Five PAs were interviewed between May and August 2014. Their experience in the role ranged from 1 to 3 years, and they occupied different positions on their personal recovery journeys. All had experienced homelessness in their recent history, accompanied by substance misuse and mental health challenges. There was also personal experience of hepatitis C infection within the sample. All participants had intimate knowledge of stigma directed against them and barriers to accessing health and social care support. All five participants were male, with an average age of 48. They have been given pseudonyms to maintain their anonymity in this paper.

From the narrative and thematic analyses, it was clear that the PAs all had different approaches to managing the client-health provider relationship. This depended on their personality, personal identity and life experience. The majority of interview dialogue focused on challenges and techniques of engaging with clients, with the health provider relationship of lesser concern. These were shared through the telling of a peer/client story. In the thematic analysis, we identified three main ‘techniques’ or strategies that PAs used to achieve ‘connectedness’ through establishment of a positive therapeutic alliance with clients: (1) rapport, (2) self-disclosure and (3) shared group membership with health services. These three themes are described below followed by a single example from the narrative analysis, which illustrates how the themes are made manifest within an individual PA’s narrative to reveal the practice of positive therapeutic alliance in their engagement with a client.

### Thematic analysis: techniques used by PAs to establish a positive therapeutic alliance

#### Rapport

Thematic analysis of the interviews revealed the building blocks of rapport and self-disclosure to precede and stimulate the development of a positive therapeutic alliance. While the PAs interviewed seemed to have difficulty expressing how they established that rapport with clients, when they were with a client they would implicitly know how to make that connection:

‘I don’t know...you...phew! there are so many ways of doing it...if you want to give me a test paper with a patient...yeah...I would know how to break him down so.’ (Steve).

The purpose of the advocate-client relationship from the perspective of Steve is revealed by this statement as he describes ‘breaking down’ the client, highlighting insight into the defence barriers erected by many individuals who have experienced adversity.

All five participants described different ways of connecting with clients to facilitate their working relationship together. Different skills and techniques became valuable at different times but respect, reciprocity and friendship were commonly used by all the PAs. Communication skills training and mentoring by more experienced colleagues within the organisation helped the PAs engage with these marginalised clients in a more professional way, by realising how to engage with them. Despite this, each PA found applying the classroom training required them to:

‘work out of our own initiative and work it out yourself.’

A frequent ice breaker was characterised by the sharing of cigarettes and offering to treat the client to breakfast/lunch/tea using the refreshment budget supplied. In exchange was the cooperation of the client to attend the appointment, this reciprocal contract provided the basis for relationship building. Upon this groundwork, it was possible to demonstrate understanding to the client and acceptance of who they are, a key marker of respect and vital quality in positive relationship formation. Within a narrative sequence of Steve’s interview, he reflects on formation of his relationship with the client. He recommends a non-hierarchical, encouraging approach that leaves the decision-making in the hands of the client. This approach is shared by all of the participants and was emphasised in their training from the Peer Advocacy Organisation:

‘you’ve got to go down by the guidelines of what is advocacy, you know, it’s client led’ (James)

‘You try and talk them into it but it’s their choice at the end of the day’ (Alan)

This engenders mutual respect and the desire to work together:

‘They are happy ‘cos they know that what they are saying is being respected’ (Bill)

Alan described specific preparations prior to the appointment so that:

‘...it’s very much, he was holding the reins’ (Alan).

In some relationships, rapport was strengthened over time through multiple inputs of the PA. As they get to know the client a bit better, they can talk about other things:

‘So you build up a friendship, just out of spending more time with them...’ (Bill)

Keeping in touch with the client was a common strategy of the advocates, sending text messages and phoning to see how they got on at the GP or at Christmas time:

‘You know just to check that he’s alright’ (Alan)

Alan further elaborates on this evolution of the client-advocate relationship by describing another client and their interaction as he displays commitment by keeping her informed of progress even if there was none:

‘I think she...at first I thought I was an annoying phone call...but I think she’s realised that, that I’m actually on her case, and a friend and a help, she’s a bit more “Oh Hello!”’

Paul elaborates on the notion of friendship:

‘I think they see me more on their level sort of thing, err, not a friend exactly but someone who’s on their team sort of thing.’

Personality seemed an important ingredient for success, rather than taught communication skills, giving the confidence to engage with sometimes challenging individuals:

‘I’m more better socialising I suppose and I think it is a more of an individual thing than it is like an overall peer model.’ (James)

Although the common technique of relationship building between the PA and the client was friendship, this is contained within a counter narrative of maintaining boundaries for the benefit of the client and the PA.

‘yeah I had to keep that boundary because you know, I know what the consequences could be’ (Steve)

I do explain to them, like you know, even though I would love to help you I just can’t, I’m not clued up to do that….you do need a barrier there for your own sanity because it is frustrating, (James)

#### Self-disclosure

Another common strategy, felt to put PAs in a unique position to help, was self-disclosure of shared experiences. Even if the experiences are not the same, sharing the experience of challenge, adversity or trauma conveys understanding to the client.

‘I’ve had drug and alcohol problems, mental health problems, so all of that helps me to empathise, you know... I can say ‘I know what you mean’ and mean it’ (Paul).

In exchange or in contrast to the earlier image of breaking down barriers, Steve would selectively share his feelings in return on the first client meeting to highlight his personal vulnerability to the client:

‘...and so suddenly he understands who I was...’

In such situations, the PA may tell of his personal experience with the aim of helping the client in giving hope to his situation, fulfilling a role model or teaching function. Selective self-disclosure is a characteristic of therapeutic alliance approaches and was used to varying extents by the PAs. This depended on their situational assessment and the impact of other rapport building skills. It confirmed their group membership with the client while facilitating relationship development

‘you can feel the tension and then you can, then you think maybe I’ll better just say oh yeah I used to be a drug addict but I had a little help I got through it, you know it is possible, something like that. Just saying that you open your hand, your cards up. It makes them trust you straight away a bit more. So you’ve got to share a bit but not too much.’ (Alan)

So I spoke with him, calmed him down, reassured him and told him everything about what I had been through and everything. He then realised that hang on this guy has been exactly the same as me and it sort of changed him. And it was just that thing, ‘Thank you, I’ll go and think about what you said’. (James)

The rationale for this disclosure was eloquently described by Alan as:

‘It’s like asking someone to fix your bike and they’ve never been on a bike before. It’s better that someone says yeah I’ve had a bike before like this.’(Alan)

In contrast, the peer advocacy organisation was described as advising the PAs not to share their past experience (Alan) but only how they are now, focusing on the restitution narrative and maintaining the client as the focus of the interaction. Alan describes the rationale for this advice as:

‘...yeah, it gives them [the client] ammunition’

This statement recognises that PAs are often still vulnerable to the context within which they are now working. Alan described how he could not work with clients in certain parts of the city due to his own recent challenging experiences there. The transition from client to PA can be very positive and supportive of the PAs development, but their experiences of substance misuse, homelessness and mental health problems remain a component of their personal narrative. The position they hold in the transition from client to advocate may fluctuate depending on personal experiences and exposure to triggers. With such a potentially vulnerable cadre, the organisation appears to promote safety in the non-disclosure of personal experiences.

However, the relationship between the PA and client creates a space for PA agency in resistance to the organisational narrative of recovery. The PAs are actively contesting the power of the organisation in their assignment of that identity label through their self-disclosure practice. However, this covert resistance could leave them vulnerable without appropriate boundary training.

#### Shared group membership

The ability of the PA to gain trust and acceptance of healthcare providers to act as a bridge to the client’s successful engagement was seen through an emphasis on shared group membership. This eased the relationship between the PA and health provider in some cases, as they were regarded as working towards the same goal. Showing a degree of flexibility by the health provider and knowing the rules of acceptable engagement by the PA facilitated attendance of the client at the appointment.

The PAs described working with health professionals in an advocacy and appointment attendance context. Awareness and understanding of health service arrangements and constraints through personal experience and training enabled the PAs to respond accordingly to achieve their aims.

Sometimes, emphasising their shared group membership was felt to facilitate quick acceptance by staff of the role of the PA and ease the working relationship:

‘...or accidently making sure they spotted the little NHS logo in the corner [of the ID badge]’. (Alan)

A story from Alan where he is advocating for his client with hostel staff shows the ability of the PA to enjoy membership of both client and staff groups. The client in this narrative has a broken door lock and believes people are stealing his belongings when he goes out. The PA tells a story of his discussion with staff who claim to see no one on the closed-circuit television entering his room. In light of this, they describe the clients’ concern as:

‘...a lot of it’s in his head’.

The PA responds to this dismissive labelling in a rational and insightful manner, exposing the lack of care, an essential group attribute, by the staff member:

‘...it’s his state of mind and so if his lock was fixed...’.

There is no reason for the PA to finish the sentence as he implies fixing the lock will ease the client’s state of mind. This insight moves him from understanding the client perspective into the therapeutic domain occupied by the worker, triggering affirmation of his position:

‘yeah well it’s been a while actually, we’ll do that yeah, sorry’.

The insertion of the apology exposes the purpose of this narrative as performing status work of the PA through this narrative of collaborative working. It illustrates an altered power dynamic in the relationship with the PA occupying the higher moral ground in his performance of group attributes. The fact that he conveys an understanding of the psyche of the client is indicative of a nurturant therapeutic alliance [24].

When staff understand the role of the PA, they work well together. This is because the PA saves them time and can help explain details of treatment or highlight issues the client mentioned earlier that may be relevant to the consultation. An example is given by Bill of working together with clinic staff to achieve attendance of the client in a way that is acceptable for the client and the clinic. Without his input, Bill feels the client would never attend due to the social disturbance caused by his condition. He feels the staff are also aware of this and so are more flexible in their approach. Bill describes arranging for the clinic receptionist to telephone him when it is their turn to see the doctor. In this way, Bill can take the client outside for a cigarette or a walk:

‘...keeping them busy and they’re not thinking about things.’

This simple example of working in collaboration with clinic staff shows how a little extra coordination and effort achieves the objective of getting the client seen.

### Narrative analysis: the use of PA techniques with a narrative structure

#### Positive therapeutic alliance

All of the interview participants used stories to illustrate how they worked with clients. An example is taken from Steve’s repertoire. It encapsulates the themes of rapport, self-disclosure and shared group membership found across the participant interviews. Moreover, it is contained within an unspoken story about development and management of a positive therapeutic alliance between himself and the client. Studying the structural composition of Steve’s story about a particularly challenging client reveals how contextual information about the world is encoded on a personal level. For example, to prepare for the impending plot of the story, a reluctant admission is made by Steve of the client’s issue with alcohol. This clearly disrupts the image Steve wishes to portray of the client, but is essential for plot progression:

‘...but there was kind of like...em...em...issue with drinking’.

However, Steve tries to claim back the image of the client:

‘...but other than that he was a perfect gentleman’

emphasising the positive regard characteristic of a therapeutic alliance as this evaluative clause positions the PA in a professional, non-judgemental role.

Steve describes the purpose of their interaction as moving the client on from his position of blaming others and anger at his liver cancer diagnosis when other people drink and do not get sick. Steve tries to rationalise with the client while maintaining hope, validating the client experience in comparison with others and remaining encouraging about the future The coherent sense of life’s sequence has been disrupted. Reconnecting the past with the present to bring some coherence is attempted by Steve:

‘and I said, “Look you’ve been drinking longer that’s why your health started, you know, getting in a bad way”...’

but these attempts are denied by the client:

‘...and all he said is blaming others...if people have a drink on the weekends how comes they don’t have this problem and why I do?’

In this scene, Steve is trying to tell the client not what he wants to hear, but what he knows already. This requires extremely sensitive communication skills, underpinned by the rapport of their relationship.

‘...because I had that kind of relationship where I could actually talk to him anyhow’.

From this close relationship and connection with the client, Steve tries to then withdraw to control the intensity of the connection. However, he may actually be trying to emphasise the connection within the narrative opportunity by threatening to withdraw. He emphasises the lack of commitment from the client when he is not available. The relationship with Steve is privileged over the importance of the medical appointment as he does not attend or rearranges if Steve is not available. Steve reports the need for the client to work with other PAs, using the advocacy organisation as a back-up for his position. He finally defaults to his professional status, acknowledging but minimising the meaning of their connection for the client by implying his contribution is a result of only doing his job.

This absolves Steve of the responsibility to reciprocate the connection:

‘so he understood’.

Steve completes the story by stepping out of the story world to give his personal evaluative comment that clients prefer the same advocate because they feel more comfortable and encouraged, as fits with a therapeutic connection. Analysis of this narrative reveals an essential component of the connection is for Steve to have agency. This agency acts to facilitate the client to accept responsibility for his situation through the therapeutic use of self. Second is the agency required to retain control of the conditions of their connection. This is illustrated through a sharing of strategies to maintain boundaries within the relationship by appealing to professionalism and organisational policy. The sub narrative of client dependence enhances the quality of the therapeutic alliance while acting in resistance to the organisational narrative of safety in minimising the opportunity for client dependence on a particular PA.

### Limitations of this study

The principle limitation of this project is the small number of participants and the specificity of their experiences. The different life stages of the PAs resulted in stronger therapeutic use of self among a selection of the participants. Structural analysis can reveal important findings related to meaning and action in narrative but can be quite technical in the reporting. Consequently, there is a heavier reporting of the thematic analysis with supportive information from the structural results to maintain the readability of this paper.

Narrative is a co-construction and the interviewer-participant relationship will always impact on the final results. As a result, reflexivity is recommended to expose the interviewer influence on the data. Both research nurses worked on the HALT: Hepatitis project. They were from an academic institution without disclosed or assumed experience of homelessness, hepatitis C or substance misuse. While they would refer clients to the PAs and receive progress updates, there was colleague familiarity without a direct health provider working relationship. There is a risk that the PAs considered the interviewers as part of the healthcare team and tailored their responses accordingly. There were numerous asides to the interviewers during the interview to orient them to the context within which the narrative was situated. This reflects an assumption that the interviewers were not aware of the ‘street’ context within which the PAs were operating. Some defensiveness may have been apparent in the client side narratives or over expression of certain behaviours, but since the focus was on peer skills in relationship building rather than details of activity or outcomes, this was able to be bracketed in the analysis.

## Discussion

Programme evaluations identify the relationship between clients and PAs as the key component of the peer support intervention’s success. The results of this project point to a more sophisticated relational dynamic in the form of a therapeutic connection, grounded in self-disclosure, as the operating principal of the PA relation. Differences in the therapist-client relationship have been shown to impact upon therapist effectiveness and exceed treatment effects [25]. Ardito and Rabellino [26] present two stages to the development of this alliance. The first stage reflects the client’s perception of the therapist as supportive. This constitutes the quality of the personal bond reflecting confidence in the role of the therapist and experience of the positive regard or non-judgemental interaction from the therapist.

PAs presented themselves as supportive and non-judgemental through the sharing of components of their story with the client. Mollica’s ‘Storytelling as a healing art’ [27] reports most traumatised people to be interested in how disclosing their story can bring hope to others. Some participants in this project told their story to clients more freely than others suggesting different purposes behind the disclosure. A storyteller is said to transfer some of their suffering to the listener suggesting a reciprocal function in the act of self-disclosure, as telling such stories can provide a healing function for an individual’s identity [27]. While self-disclosure from the advocate is often used to equalise the relationship with the client, gaining their trust and practising empathy, it can also serve to reinforce the emerging identity of the advocate and illustrate professionalism.

Self-disclosure from the PA works towards establishing equality in the relationship with the client. The fact that the PA has been in the same position as the client in terms of challenging social circumstances emphasises the peer component of the PA title. An example can be seen in an interaction described by the participant James. He clarified the conditions of his disclosure to give hope to the client and to ‘calm them down’ by acknowledging their suffering and claiming membership of their group. He felt this made a lot of difference to their relationship. Even if the experiences of the PA and client were not the same, sharing the experience of challenge, adversity or trauma conveyed understanding to the client. This understanding of the client and his situation acts as a foundation of empathic regard [24, 28–30]. This has been found to help establish rapport and promote client engagement, while encouraging the client’s own disclosure [25]. Furthermore, this understanding has been seen in experiments as a mediating process leading to client change [29]. Within this project, the components of rapport building and self-disclosure contributed to achieve the necessary relational foundation through the formation of a short-term positive therapeutic alliance [24].

If these relational techniques have been successful, Ardito and Rabellino’s [26] second stage in the development of the alliance can emerge. This reflects the collaborative relationship where client and PA share responsibility in working to achieve the goals of the interaction, such as attendance at an appointment, engagement with services or resolution of a health issue. The more experienced PAs described their relationships within these terms, showing competence to help and portraying the impression of working together [30]. Conveying confidence, hope and interest is seen as vital to facilitate the client to master their problems [31]. An issue described by one of the participants was the problem of a client’s motivation to go to the medical appointment and therefore maintain residence in the hostel. He describes his soft approach as opposed to telling the client what to do. This method returns control to the client. Furthermore, this PA’s collaborative success with the client fulfils Heinonen et al.’s [31] observation that better symptom reduction (the symptom of resistance in this situation) in clients is reported when therapists are high in friendliness and low in forcefulness. The reflection of this participant on his approach, which he feels is different from the other advocates, is a sophisticated skill in the development of professionalism [32].

The more experienced PAs described issues of dependence and preference among their clients. In contrast, the less experienced PAs appeared comfortable to allow the relationship to evolve into a friendship, with boundaries. The setting of boundaries was an important component to minimise client dependency and protect the PA’s recovery process. Regardless of the approach, there was a reliance on adaptation of taught skills to the varied and challenging interaction contexts through the use of one’s own initiative. Self-disclosure needs to be acknowledged as a key component of the therapeutic use of self in the PA’s interactions with clients. However, evidence from this project emphasises the need for support in boundary setting to protect the vulnerability of the PAs.

Balancing the relationship between the client and the health professional to facilitate patient citizenship required the advocates’ fluidity of group membership. This was enabled by their knowledge of the rules of interaction in both worlds. The PAs were able to gain the trust and acceptance of the client through self-disclosure and rapport building, and with the health team through the sharing of group attributes. This enabled the PA to act as a bridge between the two groups and facilitate positive outcomes for both. Their understanding of service constraints and sharing of certain episode objectives with the health team facilitated a collaborative style of working together. The PAs showed significant levels of self-awareness when describing successes in these relationships.

Framing the peer advocacy model within the literature of therapeutic use of self provides a credible theoretical underpinning to their unique model of practice. It offers a moral ground for self-disclosure in relationship building. Consequently, the evidence from this project has charted the positive impact of selective self-disclosure and non-hierarchical approach for rapport building. These can be seen as key components of the therapeutic use of self that effect positive outcomes.

## Conclusion

Practical recommendations from this project include self-esteem work among new PAs to facilitate their confidence to engage with clients. This could include communication and advocacy skills sessions. Within this communication domain is the need for support from the PA organisation in the safe use of self-disclosure with guidance in boundary setting in client relationships. This is in opposition to the denial of this unique attribute of the PA’s activity. A structured mentorship model could act as the link between classroom learning and its practical application once working with clients.

To enhance the PA-health provider relationship, there should be awareness raising of the function of PAs among healthcare staff in their trust or practice orientations. PAs should be issued with NHS identity badges to provide a visible and quick sign of shared group membership with the health providers. A long-term aim stimulated by the findings of this project is an NHS/Clinical Commissioning Group accreditation of PAs to give credibility to their role in the health and social services. This would also raise awareness among practitioners of their unique and valuable model of practice.

## Abbreviations

PA: Peer advocate
